# Ectopic Pregnancy

**Published:** 1906-03

**Authors:** Henry K. Leake

**Affiliations:** Professor Gynecology Med. Department S. W. University, Dallas, Texas


For Texas Medical Journal.

### Ectopic Pregnancy.

BY HENRY K. LEAKE, M. D., PROFESSOR GYNECOLOGY MED. DEPARTMENT S. W. UNIVERSITY, DALLAS, TEXAS.

*Editor of Texas Medical Journal.*

The discussion that followed the reading of my paper on the "Treatment of Ectopic Pregnancy"* before the North Texas Medical Association last June, emphasized the importance of making the diagnosis of tubal pregnancy before rupture. Since that meeting I have had two additional cases where this was done and followed by operation with speedy recovery of both patients.

---

*The paper was published in the *Texas State Journal of Medicine* in October, 1905, by vote of the North Texas Medical Society.

The first patient, a large woman with fat and tense abdominal walls, had been under the treatment of her family physician for "threatened abortion," but this diagnosis was corrected by a more careful examination.

The second patient was one of Dr. Leslie Moore's (of Van Alstyne, Texas), and it is to his skillful diagnosis and insistence upon timely operation, that the woman owes her life. In this case, it is noteworthy, also, that several years previously a moderate-sized blood mass appeared in the pelvis that was aspirated *per vaginam* by another physician who had charge of the case. Presumably this haematocele was the result of a small rupture of the tube, or more probably a tubal abortion, the blood gravitating into Douglass' pouch and coming from the right side, for, at the second operation, which exsected the left appendage, containing the gestation product, adhesions were found, binding down the right appendage to neighboring structures; these adhesions were broken up during the operation.

In these patients the gestation sac was on the point of rupture that possibly could not have been delayed longer than a few days. At some risk they were transferred to my private hospital at Dallas, where the operations were done with the very best surroundings. However, the greatest care was taken in their removal.

